# Supplementary figures and images for: The TMEM192-mKeima probe specifically assays lysophagy and reveals its initial steps
Source: J Cell Biol. 2023 Oct 6;222(12):e202204048. doi: 10.1083/jcb.202204048 (PMC10558291; doi:10.1083/jcb.202204048)

Figure 4F

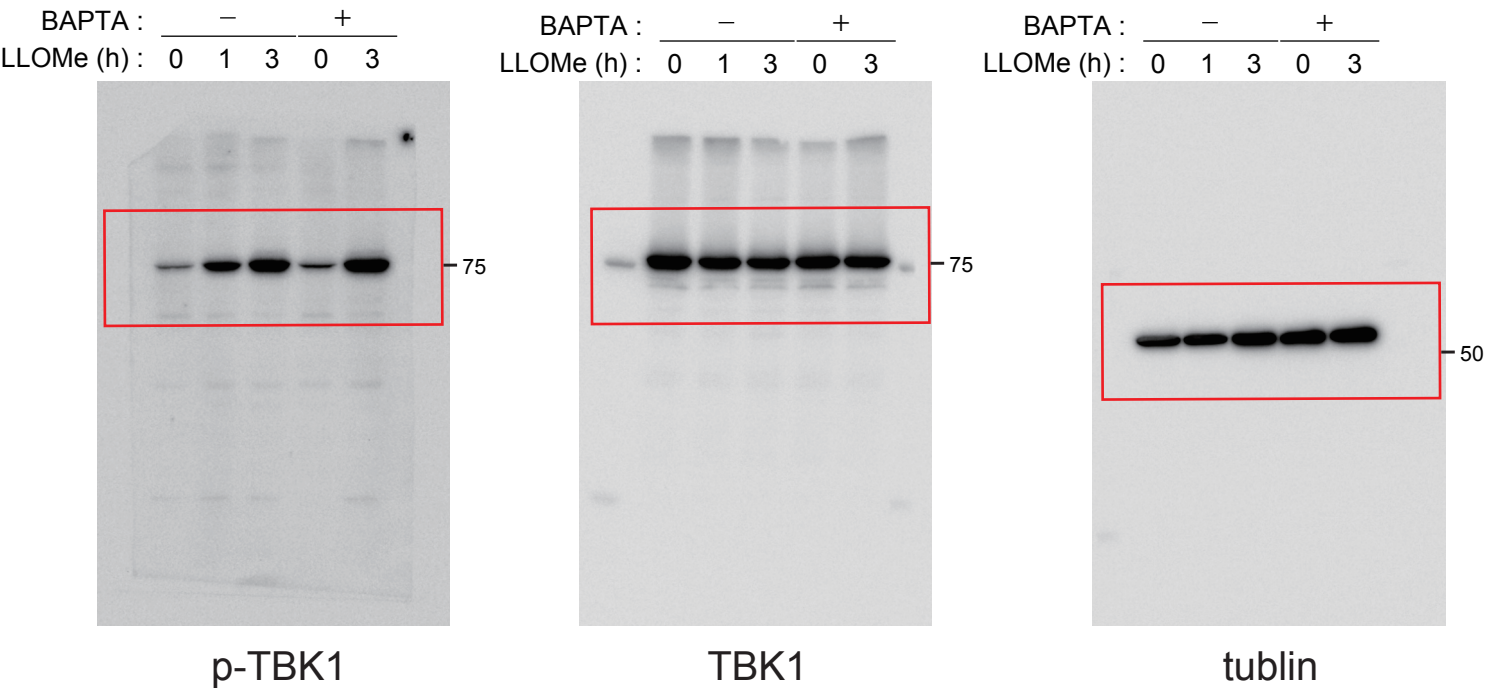

Supplement: SourceData F4 — is the source file for Fig. 4. [file JCB_202204048_SourceDataF4.pdf]
